# Cancer mortality in 13 to 29-year-olds in England and Wales, 1981–2005

**DOI:** 10.1038/sj.bjc.6604080

**Published:** 2007-11-06

**Authors:** M Geraci, J M Birch, R D Alston, A Moran, T O B Eden

**Affiliations:** 1 Cancer Research UK Paediatric and Familial Cancer Research Group, School of Cancer and Imaging Sciences, University of Manchester, Stancliffe, Hospital Road, Manchester M27 4HA, UK; 2 North West Cancer Intelligence Service, Christie Hospital NHS Trust, Withington, Kinnaird Road, Manchester M20 4QL, UK; 3 Teenage Cancer Trust Young Oncology Unit, School of Cancer and Imaging Sciences, University of Manchester, Christie Hospital NHS Trust, Withington, Wilmslow Road, Manchester M20 4BX, UK

**Keywords:** teenage and young adult cancer, mortality trends, cancer services, central nervous system tumours, haematological malignancies, bone tumours

## Abstract

We examined cancer mortality at ages 13–29 years in England and Wales between 1981 and 2005, a total of 20 026 deaths over approximately 303 million person-years (mpy) at risk by sex, age group and time period. Overall, the mortality rate was 65.6 per mpy. Malignant neoplasms of the central nervous system showed the highest rate (8.5), followed by myeloid and monocytic leukaemia (6.6), lymphoid leukaemia (6.4), malignant bone tumours (5.4) and non-Hodgkin's lymphoma (5.2). These groups together accounted for almost 50% of all cancer deaths. The mortality rate for males (72.4) was 23% higher than for females (58.6) (*P*-value <0.0001). Males showed significantly higher mortality rates than females in almost all diagnostic groups, in general, mortality increasing with age (*P*-value <0.0001). There were significant decreases in mortality over time, the annual percentage change between 1981 and 2005 being minus 1.86 (95% confidence interval −2.09 to −1.62). Cancer groups with the highest mortality differed from those with the highest incidence.

One in four of all deaths in the United Kingdom is caused by cancer (Coleman *et al*, 1999). Although the majority of these deaths occur in the elderly, cancer represents the most common cause of death at ages 13–29 years. Approximately 2500 teenagers and young adults (TYAs) died from cancer in England and Wales in the 4-year period 2002–2005. Little is known about the aetiology of cancers in such young people, though; it is likely that it differs from that in older adults (Birch, 2005, pp 13–31).

We carried out detailed analyses of mortality rates by cancer group, sex, age and time period in persons aged 13–29 years in England and Wales, to identify the highest mortalities and lowest reductions in mortality over time; these, it is hoped, will assist with targeting of clinical resources and service planning.

## METHODS

Mortality data on neoplasms in England and Wales from 1981 to 2005 were provided by the Office for National Statistics, London (ONS); those covered malignant neoplasms, benign tumours and neoplasms of uncertain behaviour, in those aged 13–29 years. For each observation, age at death, sex, and diagnosis coded according to the International Classification of Diseases (ICD) were included. Data on cases registered from 1981 to 2001, a period that spans both the ICD Ninth Revision (ICD-9) (World Health Organization, 1977) and ICD Tenth Revision (ICD-10) (World Health Organization, 1992) coding epochs at the ONS, were released by the English Cancer Information System (Office for National Statistics, 2004) along with the tabulation for the conversion from the earlier ICD-9 to ICD-10. Direct translation between ICD-9 and ICD-10 at the 4-digit level for all codes was not possible. We, therefore, used the ICD-10 codes up to the third digit. For comparison purposes, we also considered incidence data on all registered neoplasms diagnosed in England from 1979 to 2003 inclusive, supplied by the National Cancer Intelligence Centre, ONS, London (Office for National Statistics, 2006b).

Mid-year estimates, by single year of age and sex, of the resident population in England and Wales for the time period 1981–2005 were obtained, based on the national censuses (Office for National Statistics, 2007). Number of deaths and population estimates were tabulated by age, sex and time period. The age groups were 13–14, 15–19, 20–24 and 25–29 years. The time span was divided into five quinquennia. Thirteen main diagnostic groups were defined as shown in Table 1, three of which were further subclassified giving 14 groups in all and for each, mortality rates were calculated. The European standard population was used for direct standardisation of the rates (Quinn *et al*, 2005). Throughout this report rates are given per million person-years (mpy).

Heterogeneity between sexes, age groups, and time periods was tested for by using a *χ*^2^ test statistic. Age and temporal trends was tested for, by using a single degree of freedom *χ*^2^ statistic (Armitage, 1955); the estimate of the percent change per year of age (PCYA) was obtained by using a linear regression of the natural logarithm of the rates; similarly, in the temporal analysis the annual percent change (APC) was estimated by using a weighted linear regression of the natural logarithm of the age-adjusted rates where the weights were given by the inverse of the estimated variance of the response variable (Kim *et al*, 2000). Significance level was set at 5%.

The results of the separate stratified analyses were successively assessed with Poisson additive models (Wood, 2006), including main effects, interaction terms, and non-linear temporal trends simultaneously. The statistical analyses were performed using Stata v. 9.2 (StataCorp, 2005) and the software R (R Development Core Team, 2006).

## RESULTS

A total of 20 026 deaths were registered over 25-year period in people aged 13–29 years with ICD-10 codes C00-D48, of which 875 were from benign, uncertain or unknown behaviour tumours. There were approximately 303 million person-years at risk. Overall, the mortality rate was 65.6 per mpy (Table 2). Malignant tumours of the central nervous system (CNS) showed the highest mortality rate (8.5), followed by myeloid and monocytic leukaemia (MML) (6.6), lymphoid leukaemia (LL) (6.4), malignant bone tumours (bone) (5.4) and non-Hodgkin's lymphoma (NHL) (5.2). These groups together accounted for almost 50% of all deaths under study.

Overall, the mortality rate for males (72.4) was 23% higher than for females (58.6), and significantly higher in all the specified groups except breast and genitourinary (GU) organs, where females had higher rates; there was no significant heterogeneity by sex for liver cancer, melanoma, GU sites other than gonads and cervix, thyroid and non-malignant neoplasms of the CNS. There were large disproportions of rates between males and females for cancers of the lip, oral cavity and pharynx, respiratory organs, bone and lymphomas and leukaemias. In these groups, the ratio of rates in males compared to females was between 1.5 and 2.2.

The mortality differed between age groups for all specified diagnostic groups except thyroid and other endocrine glands (Table 3). The rates for breast and cervical cancer increased by 50% for each additional year of age. Marked increases across age groups were also observed for digestive organs (excluding liver), melanoma of skin and testicular cancer, whereas for bone cancer decreases on average by nearly 8% per year of age, the largest drop among the diagnostic groups. Lymphomas and MML rates increased rates with age, with the largest increases at ages 13–19 years. Mortality for LL peaked at age 15–19 years. For Hodgkin's lymphoma (HL), mortality for persons aged 25–29 years was 11 times higher than the rate for those aged 13–14 years.

When using the Poisson regression approach, few groups showed a significant interaction between sex and age. For bone, the relative risk in males compared to females increased with age, from 0.8 (95% confidence interval, CI, 0.6–1.1) at 13–14 years to 2.1 (95% CI 1.6–2.8) at 25–29 years. For NHL, the relative risk decreased by age, in males being three (95% CI 1.7–5.3) times higher at 13–14 years and 1.7 (95% CI 1.4–2) times higher at 25–29 years.

Mortality for all cancers combined decreased over time by 1.86% on average each year (Table 4). Numbers decreasing from about 1000 per year in 1981–1985 to a little over 600 per year in 2001–2005 period. Testicular cancer showed the largest APC (−6.31), followed by HL (−5.12), cancer of respiratory and intra-thoracic organs (−4.26), and cervical cancer (−4.07). Neither the total nor the sex-specific mortality rates for bone showed a significant trend over time. Further analyses revealed that the rates, after decreasing in the first three time periods, increased in the last 10 years at age 20–24 years (APC 1.43, CI 0.16–2.72); this was also supported by the Poisson regression.

Groups with the highest mortality (CNS, MML, LL, bone, NHL) differed from those with the highest incidence (HL, testis, melanoma, CNS, cervix) (Table 5). CNS, MML, LL, bone and soft tissue were all in the top 10 for mortality and were between 3 and 8 rank places higher than for incidence. The ratio of incidence to mortality varied from 16 : 1 for testis and 11 : 1 for thyroid cancer to where the rates are similar, as for liver. The ratio for MML, LL, and bone was below 2 : 1. Testicular and cervical cancer, melanoma, and HL were among the top five groups with the highest incidence between 1979 and 2003 but below the 5th position for mortality between 1981 and 2005, with deaths numbering much below that of new cases.

In 2002–2005, approximately 2500 TYAs in England died from neoplasms, of which 2341 were coded as malignant, making cancer the commonest disease-related cause of death at these ages, accounting for approximately 12% of all deaths and exceeded only by deaths from transport accidents, though at 13–14 years neoplasmic deaths outnumbered deaths from any other cause (Table 6). Deaths from all causes by sex and age group showed a heterogeneous distribution.

Among the 875 deaths coded as being due to benign (275) or uncertain and unknown behaviour (600) neoplasms, 166 involved endocrine glands, 49 middle ear, respiratory and intrathoracic organs, 57 mesothelial and soft tissue, 31 haemopoietic and 23 of GU organs and 549 other sites.

## DISCUSSION

This is the first study to present detailed analyses of cancer mortality in TYAs in England and Wales and makes use of recent national data over a 25-year period. The introduction of ICD-10 in 2001 had an impact on the analysis of trends in cancer mortality (Brock *et al*, 2004). Comparability ratios provide a measure of the effect of changes in coding practice that may vary by site and age. Approximated estimates of variations in rates of TYA mortality trends showed substantial robustness of the data to the introduction of the ICD-10 coding system. The results demonstrate that cancer mortality in TYAs has decreased over time. Yet, cancer is still the most common disease-related cause of death in this age group. Moreover, the sites of most lethal cancers differ from those at higher ages (Office for National Statistics, 2006a). Some of the deaths due to benign neoplasms or those of uncertain behaviour may have been miscoded or reported inaccurately. However, the small proportion of deaths so assigned to ill-defined sites supports the robustness of the data.

Major mortality reductions occurred for cancers of (i) respiratory and intrathoracic organs, mainly bronchus and lung, (ii) GU organs, cervical and testicular cancer in particular, and (iii) HL; whereas cancers of digestive organs, soft tissue, and CNS showed lower decreases in rates and less favourable incidence to mortality ratios. There were no corresponding incidence falls of these cancers in TYAs and in contrast some temporal increases have been noted (Birch, 2005). The marked reductions in mortality must therefore be due to well-documented improvements in survival, particularly from testis cancer and HL (Coleman *et al*, 1999). Given our study period, it is likely that cervical screening will have contributed to the reduction in cervix cancer mortality. That from melanoma remained relatively constant over time, in spite of an increase in incidence, and was higher in males. In contrast, incidence was higher in females indicating that survival for melanoma is higher in females and has increased over time. Increased public and physician awareness of melanoma in young people may have contributed to these changes.

Previous national studies of cancer mortality in England and Wales presented data up to 1997 (Swerdlow *et al*, 2001) and 2003 (Quinn *et al*, 2001; Rowan *et al*, 2005) and included figures for broad and variable age groups (e.g. 15–34, 20–44) depending on cancer site. Furthermore, not all relevant cancer sites were reported in detail for example bone, soft tissue, thyroid, HL, MML, LL and liver (Quinn *et al*, 2001); bone, soft tissue, MML, LL (Swerdlow *et al*, 2001), thereby preventing direct comparisons. Levi *et al* (2003) reported mortality trends at 15–24 years in Europe, including the United Kingdom but compared, 1965–1969 with 1995–1998, and only for selected diagnostic groups (bone, soft tissue, testis, NHL, HL, and all leukaemias combined), again, precluding direct comparison.

A recent study from the United States covering the period 1975–2000 included some mortality at ages 15–29 years (Bleyer *et al*, 2006), but was primarily on incidence and survival. In general, the trends were compatible with the present study though some differences were apparent. Thus, decreases in mortality at 15–29 years were reported for melanoma, colorectal carcinoma and testicular cancers; there were smaller reductions for CNS tumours and breast cancer. For liver cancer there was an overall decrease, but mortality increased in the more recent years, but time trends for other groups were not reported. In the present study, up to year 2000, no reduction in mortality from colorectal cancer was seen, but there was a steady decrease in breast cancer and mortality from liver cancer was stable. These differences may, at least in part, be due to differences in the ethnic mix in the United States, England, and Wales.

The recent guidance on improving outcomes in children and young people with cancer, published by the National Institute for Health and Clinical Excellence (National Collaborating Centre for Cancer, 2005) noted the lack of available comprehensive data on cancers in TYAs. The present report provides this for mortality in England and Wales. The results highlight diagnostic groups, which present the greatest clinical challenges, including those in which the ratio of incidence to mortality is low, indicating poor survival and those groups with little or no reductions in mortality over time. The four leading causes of cancer death, CNS tumours, MML, LL, and bone cancers, all have low incidence to mortality ratios. Furthermore, over the 25-year study period, mortality in CNS and bone tumours has decreased by less than 1%. These observations may assist with resource allocation and the setting of clinical targets.

## Figures and Tables

**Table 1:** Diagnostic groups based on the ICD-10 codes used for the analysis of the mortality data of persons aged 13–29 years in England and Wales from 1981 to 2005

**Description**		
**Main group**	**Subgroup**	**ICD-10 codes**
Lip, oral cavity and pharynx		C00–C14
Digestive organs		C15–C26
	Colorectal	C18, C19, C20
	Liver	C22
	Other sites in GI tract	C15–C17, C21, C23–C26
		
Respiratory and intra-thoracic organs		C30–C39
Bone and articular cartilage		C40–C41
Melanoma of skin		C43
Mesothelial and soft tissue		C45–C49
Breast		C50
Genitourinary organs		C51–C68
	Cervix	C53
	Ovary	C56
	Testis	C62
	Other sites	C51, C52, C54, C55, C57–C61, C63–C68
		
Eye, brain, and other parts of CNS		C69–C72
Thyroid and other endocrine glands		C73–C75
Malignant neoplasms of lymphoid, haematopoietic, and related tissue		C81–C96
	Lymphoid leukaemia	C91
	Myeloid and monocytic leukaemia	C92, C93
	Hodgkin's lymphoma	C81
	Non-Hodgkin's lymphoma	C82, C83, C84, C85
	Other and unspecified lympho-haematopoietic	C88, C90, C94, C95, C96
		
Other malignant neoplasms		C44, C76–C80, C97
Benign neoplasms and neoplasms of uncertain behaviour		D10-D48
	Eye, brain, and other parts of CNS	D31, D32, D33, D42, D43
	Other sites	D10-D30, D34-D41, D44-D48

**Table 2:** Number of deaths (*N*) and mortality rates (*R*) per million person–years at risk by sex and diagnostic group at ages 13–29 years in England and Wales, 1981–2005

		**Male**		**Female**		**Persons**		***P*-value**
**Main group**	**Subgroup**	***N***	***R***	***N***	***R***	***N***	***R***	**Heter.**
Lip, oral cavity and pharynx		194	1.3	98	0.6	292	1.0	<0.0001
Digestive organs		765	4.9	571	3.7	1336	4.3	<0.0001
	Colorectal	300	1.9	206	1.4	506	1.6	<0.0001
	Liver	164	1.1	148	1.0	312	1.0	0.4268
	Other sites in GI tract	301	1.9	217	1.4	518	1.7	0.0003
Respiratory and intrathoracic organs		257	1.7	162	1.1	419	1.4	<0.0001
Bone and articular cartilage		993	6.5	632	4.3	1625	5.4	<0.0001
Melanoma of skin		420	2.7	364	2.4	784	2.5	0.0526
Mesothelial and soft tissue		620	4.0	462	3.1	1082	3.5	<0.0001
Breast		1	0.0	719	4.6	720	2.3	<0.0001
Genitourinary organs		783	5.1	1376	8.9	2159	7.0	<0.0001
	Cervix	—	—	773	5.0	773	5.0	—
	Ovary	—	—	410	2.7	410	2.7	—
	Testis	621	4.0	—	—	621	4.0	—
	Other sites	162	1.0	193	1.3	355	1.2	0.0806
Malignant neoplasms of CNS		1531	10.0	1068	7.1	2599	8.5	<0.0001
Thyroid and other endocrine glands		150	1.0	139	0.9	289	1.0	0.6136
Malignant neoplasms of lymphoid, haematopoietic and related tissue		4410	28.7	2610	17.3	7020	23.1	<0.0001
	Lymphoid leukaemia	1308	8.6	620	4.2	1928	6.4	<0.0001
	Myeloid and monocytic leukaemia	1154	7.5	844	5.6	1998	6.6	<0.0001
	Hodgkin's lymphoma	762	4.9	516	3.4	1278	4.2	<0.0001
	Non-Hodgkin's lymphoma	1037	6.7	548	3.6	1585	5.2	<0.0001
	Other and unspecified lympho-haematopoietic	149	1.0	82	0.6	231	0.8	<0.0001
Other malignant neoplasms		522	3.4	304	2.0	826	2.7	<0.0001
Benign neoplasms and neoplasms of uncertain behaviour		489	3.2	386	2.6	875	2.9	0.0011
	Eye, brain, and other parts of CNS	204	1.3	184	1.2	388	1.3	0.3823
	Other sites	285	1.9	202	1.4	487	1.6	0.0003
All groups		11 135	72.4	8891	58.6	20 026	65.6	<0.0001

**Table 3:** Number of deaths (*N*) and mortality rates (*R*) per million person–years at risk by age and diagnostic group at ages 13–29 years in England and Wales, 1981–2005

		**13–14**		**15–19**		**20–24**		**25–29**		**P-value**	**PCYA^a^**	
**Main group**	**Subgroup**	***N***	***R***	***N***	***R***	***N***	***R***	***N***	***R***	**Heter.**	**Value**	**(95% CI)**
Lip, oral cavity and pharynx		19	0.6	68	0.8	88	1.0	117	1.3	0.0006	5.52^*^	(2.82,8.30)
Digestive organs		26	0.8	147	1.7	358	4.0	805	8.7	<0.0001	18.71^*^	(17.47,19.97)
	Colorectal	8	0.2	46	0.5	131	1.4	321	3.5	<0.0001	21.97^*^	(18.19,25.88)
	Liver	14	0.4	66	0.8	99	1.1	133	1.4	<0.0001	8.03^*^	(5.87,10.25)
	Other sites in GI tract	4	0.1	35	0.4	128	1.4	351	3.8	<0.0001	29.03^*^	(24.33,33.90)
Respiratory and intrathoracic organs		10	0.3	48	0.6	111	1.2	250	2.7	<0.0001	17.66^*^	(14.82,20.57)
Bone and articular cartilage		199	6.0	673	7.8	503	5.6	250	2.7	<0.0001	−7.63^*^	(−10.43,−4.75)
Melanoma of skin		7	0.2	60	0.7	239	2.6	478	5.2	<0.0001	27.63^*^	(21.44,34.13)
Mesothelial and soft tissue		71	2.1	282	3.3	361	4.0	368	4.0	<0.0001	3.75^*^	(1.63,5.91)
Breast		0	0.0	9	0.1	72	0.8	639	6.9	<0.0001	49.81^*^	(44.42,55.42)
Genitourinary organs		32	1.0	186	2.2	562	6.2	1379	14.9	<0.0001	22.21^*^	(20.42,24.02)
	Cervix	0	0.0	6	0.1	123	2.7	644	13.9	<0.0001	54.12^*^	(47.02,61.56)
	Ovary	11	0.7	58	1.4	124	2.8	217	4.7	<0.0001	14.67^*^	(11.83,17.59)
	Testis	2	0.1	73	1.7	216	4.8	330	7.1	<0.0001	26.51^*^	(17.88,35.77)
	Other sites	19	0.6	49	0.6	99	1.1	188	2.0	<0.0001	11.85^*^	(8.72,15.07)
Malignant neoplasms of CNS		260	7.8	570	6.6	721	8.0	1048	11.3	<0.0001	3.85^*^	(2.03,5.71)
Thyroid and other endocrine glands		29	0.9	82	0.9	83	0.9	95	1.0	0.8513	0.91	(−1.64,3.52)
Malignant neoplasms of lymphoid, haematopoietic and related tissue		543	16.2	1864	21.6	2129	23.5	2484	26.8	<0.0001	3.13^*^	(2.05,4.23)
	Lymphoid leukaemia	261	7.8	768	8.9	536	5.9	363	3.9	<0.0001	−6.36^*^	(−8.17,−4.52)
	Myeloid and monocytic leukaemia	147	4.4	478	5.5	616	6.8	757	8.2	<0.0001	4.50^*^	(3.75,5.27)
	Hodgkin's lymphoma	20	0.6	207	2.4	451	5.0	600	6.5	<0.0001	15.88^*^	(11.04,20.94)
	Non-Hodgkin's lymphoma	78	2.3	340	3.9	466	5.2	701	7.6	<0.0001	7.97^*^	(6.52,9.43)
	Other and unspecified lympho-haematopoietic	37	1.1	71	0.8	60	0.7	63	0.7	0.0803	−2.12	(−6.45,2.41)
Other malignant neoplasms		27	0.8	135	1.6	218	2.4	446	4.8	<0.0001	13.80^*^	(10.97,16.69)
Benign neoplasms and neoplasms of uncertain behaviour		89	2.7	199	2.3	250	2.8	337	3.6	<0.0001	3.36^*^	(2.02,4.72)
	Eye, brain, and other parts of CNS	42	1.3	78	0.9	105	1.2	163	1.8	<0.0001	4.58^*^	(1.19,8.08)
	Other sites	47	1.4	121	1.4	145	1.6	174	1.9	0.0526	2.41^*^	(0.91,3.93)
All groups		1312	39.2	4323	50.0	5695	63.0	8696	93.8	<0.0001	6.53^*^	(5.80,7.26)

a Percentage change per year of age.

^*^Denotes significance at the 5% level.

**Table 4:** Number of deaths (*N*) and mortality rates (*R*) per million person–years at risk by time period and diagnostic group at ages 13–29 years in England and Wales, 1981–2005

		**1981–1985**		**1986–1990**		**1991–1995**		**1996–2000**		**2001–2005**		**P-value**		**APC^a^**	
**Main group**	**Subgroup**	***N***	***R***	***N***	***R***	***N***	***R***	***N***	***R***	***N***	***R***	**Heter.**	**Trend**	**value**	**(95% CI)**
Lip, oral cavity and pharynx		63	1.0	69	1.0	53	0.9	57	1.0	50	0.9	0.7776	0.5251	−0.54	(−1.64,0.56)
Digestive organs		298	4.8	317	4.8	253	3.9	247	4.2	221	3.9	0.0226	0.0053	−0.92^*^	(−1.82,0.00)
	Colorectal	116	1.9	120	1.8	82	1.2	104	1.8	84	1.5	0.0330	0.1086	−0.41	(−1.87,1.06)
	Liver	66	1.0	71	1.1	58	0.9	52	0.9	65	1.1	0.6814	0.9109	−0.13	(−1.86,1.63)
	Other sites in GI tract	116	1.9	126	1.9	113	1.7	91	1.5	72	1.3	0.0466	0.0028	−1.66^*^	(−3.05,−0.25)
Respiratory and intrathoracic organs		139	2.3	90	1.4	74	1.1	63	1.1	53	0.9	<0.0001	<0.0001	−4.26^*^	(−5.56,−2.94)
Bone and articular cartilage		414	6.2	331	5.2	280	4.8	280	5.1	320	5.6	0.0104	0.1444	−0.49	(−1.31,0.34)
Melanoma of skin		167	2.7	165	2.4	183	2.8	134	2.3	135	2.4	0.3428	0.2319	−0.64	(−1.69,0.43)
Mesothelial and soft tissue		231	3.6	258	3.9	232	3.8	190	3.4	171	3.0	0.0648	0.0339	−0.86	(−1.76,0.05)
Breast		176	3.0	174	2.6	151	2.2	127	2.0	92	1.6	<0.0001	<0.0001	−2.62^*^	(−3.48,−1.75)
Genitourinary organs		623	10.1	560	8.4	412	6.2	311	5.2	253	4.5	<0.0001	<0.0001	−4.24^*^	(−4.90,−3.56)
	Cervix	207	7.0	199	5.9	159	4.6	115	3.7	93	3.3	<0.0001	<0.0001	−4.07^*^	(−5.20,−2.92)
	Ovary	116	3.7	87	2.6	85	2.6	70	2.4	52	1.8	0.0003	<0.0001	−2.80^*^	(−4.02,−1.57)
	Testis	207	6.6	183	5.4	106	3.3	75	2.6	50	1.8	<0.0001	<0.0001	−6.31^*^	(−7.48,−5.11)
	Other sites	93	1.5	91	1.4	62	1.0	51	0.9	58	1.0	0.0039	0.0009	−2.60^*^	(−4.10,−1.07)
Eye, brain, and other parts of CNS		559	8.8	609	9.4	534	8.7	435	7.6	462	8.1	0.0170	0.0141	−0.74^*^	(−1.47,−0.01)
Thyroid and other endocrine glands		62	1.0	64	1.0	70	1.2	50	0.9	43	0.8	0.2553	0.2294	−0.77	(−3.11,1.62)
Lymphoid, haematopoietic and related tissue		1858	28.8	1636	25.1	1461	23.8	1106	19.7	959	16.9	<0.0001	<0.0001	−2.57^*^	(−2.93,−2.22)
	Lymphoid leukaemia	469	7.1	463	7.3	385	6.6	346	6.3	265	4.6	<0.0001	<0.0001	−1.81^*^	(−2.62,−0.99)
	Myeloid and monocytic leukaemia	555	8.6	463	7.1	387	6.3	312	5.5	281	4.9	<0.0001	<0.0001	−2.70^*^	(−3.42,−1.97)
	Hodgkin's lymphoma	397	6.3	335	5.0	270	4.2	139	2.4	137	2.4	<0.0001	<0.0001	−5.12^*^	(−6.07,−4.15)
	Non-Hodgkin's lymphoma	362	5.6	331	5.0	382	6.0	271	4.8	239	4.2	<0.0001	0.0008	−1.20^*^	(−2.11,−0.28)
	Other and unspecified	75	1.2	44	0.7	37	0.6	38	0.7	37	0.6	0.0029	0.0034	−3.31^*^	(−5.26,−1.32)
Other malignant neoplasms		166	2.6	141	2.1	195	3.0	166	2.8	158	2.8	0.0189	0.1097	0.46	(−0.81,1.75)
Benign and uncertain behaviour		235	3.7	145	2.2	172	2.9	181	3.2	142	2.5	<0.0001	0.0435	−1.02	(−2.35,0.32)
	Eye, brain, and other parts of CNS	112	1.8	61	0.9	75	1.2	73	1.3	67	1.2	0.0012	0.0690	−1.61	(−3.50,0.32)
	Other sites	123	1.9	84	1.3	97	1.6	108	1.9	75	1.3	0.0075	0.2782	−0.38	(−1.82,1.09)
All groups		4991	78.4	4559	69.5	4070	65.2	3347	58.5	3059	53.9	<0.0001	<0.0001	−1.86^*^	(−2.09,−1.62)

a Annual percentage change.

^*^Denotes significance at the 5% level.

**Table 5:** Ranking of selected diagnostic groups based on the number (*N*) of deaths from 1981 to 2005 and ranking based on the number of new cases registered in England from 1979 to 2003 in persons at 13–29 years

	**Mortality**			**Incidence**			
**Group**	***N***	**%** **a**	**Rank**	***N***	**%** **b**	**Rank**	**I:M ratio** **c**
Eye, brain, and other parts of CNS	2473	13.9	1	6003	8.2	4	2.4
Myeloid and monocytic leukaemia	1899	10.7	2	3095	4.2	8	1.6
Lymphoid leukaemia	1843	10.4	3	2663	3.7	11	1.4
Bone and articular cartilage	1538	8.7	4	2760	3.8	10	1.8
Non-Hodgkin's lymphoma	1513	8.5	5	4873	6.7	6	3.2
Hodgkin's lymphoma	1209	6.8	6	9778	13.4	1	8.1
Mesothelial and soft tissue	1019	5.7	7	2539	3.5	13	2.5
Melanoma of skin	731	4.1	8	6744	9.3	3	9.2
Cervix	713	4.0	9	5520	7.6	5	7.7
Breast	683	3.9	10	3874	5.3	7	5.7
Testis	590	3.3	11	9757	13.4	2	16.5
Other sites in GI tract	487	2.7	12	819	1.1	19	1.7
Colorectal	485	2.7	13	1511	2.1	16	3.1
Respiratory and intrathoracic organs	402	2.3	14	1051	1.4	18	2.6
Ovary	376	2.1	15	2417	3.3	14	6.4
Benign and uncertain behaviour - CNS	373	2.1	16	2627	3.6	12	7.0
Other sites in GU organs	338	1.9	17	1762	2.4	15	5.2
Liver	295	1.7	18	389	0.5	20	1.3
Lip, oral cavity, and pharynx	276	1.6	19	1254	1.7	17	4.5
Thyroid and other endocrine glands	272	1.5	20	3063	4.2	9	11.3
Other and unspecified lympho-haematopoietic	222	1.3	21	381	0.5	21	1.7
All groups	17 737	100		72 880	100		4.1

a Percentage of all deaths.

b Percentage of all new cases.

c Incidence to mortality ratio; number of new cases divided by number of deaths.

**Table 6:** Numbers (*N*) and sex ratios (M:F) by age group for the 10 leading causes of death and all deaths at ages 13–29 years in England and Wales, 2002–2005

	**13–14**		**15–19**		**20–24**		**25–29**		**13–29**	
**Cause of death**	***N***	**M:F**	***N***	**M:F**	***N***	**M:F**	***N***	**M:F**	***N***	**M:F**
Transport accidents	138	2.7	1441	3.7	1538	5.2	1011	5.2	4128	4.5
Neoplasms	187	1.4	595	1.2	722	1.3	964	1.0	2468	1.2
Intentional self-harm	15	0.9	331	3.1	864	4.3	1113	3.6	2323	3.7
Diseases of the circulatory system	52	1.4	277	1.6	392	1.5	643	2.0	1364	1.7
Mental and behavioural disorders	18	1.2	157	2.1	479	4.4	709	5.0	1363	4.1
Diseases of the nervous system	101	1.5	422	2.2	402	1.8	398	1.6	1323	1.8
Diseases of the respiratory system	55	1.4	133	1.3	192	1.1	222	1.6	602	1.3
Endocrine, nutritional, and metabolic diseases	36	1.8	134	0.8	208	1.3	193	1.1	571	1.1
Malformations, deformations, and abnormalities	54	1.2	157	1.5	165	1.4	168	1.0	544	1.3
Diseases of the digestive system	14	0.6	64	1.2	129	1.3	265	1.8	472	1.5
Other causes	227	1.5	1292	2.4	1965	2.9	2259	2.7	5743	2.6
All deaths	897	1.5	5003	2.2	7056	2.7	7945	2.4	20 901	2.4

## References

[bib1] Armitage P (1955) Tests for linear trends in proportions and frequencies. Biometrics 11: 375–386

[bib2] Birch JM (2005) Patterns of incidence of cancer in teenagers and young adults: implications for Aetiology. In: Eden TOB, Barr RD, Bleyer A, Whiteston M (eds) Cancer and the Adolescent 2nd edn, London: Blackwell Publishing, pp 13–31

[bib3] Bleyer A, O'Leary M, Barr R, Ries LAG (eds) (2006) Cancer Epidemiology in Older Adolescents and Young Adults 15–29 Years of Age, Including SEER Incidence and Survival: 1975–2000. Bethesda, MD: National Cancer Institute, NIH

[bib4] Brock A, Griffiths C, Rooney C (2004) The effect of the introduction of ICD-10 on cancer mortality trends in England and Wales. Office for National Statistics, Health Statistics Quarterly 23: 7–17 15704380

[bib5] Coleman MP, Babb P, Damiecki P, Grosclaude P, Honjo S, Jones J, Knerer G, Pitard A, Quinn M, Sloggett A, De Stavola B (1999) Cancer Survival Trends in England and Wales, 1971–1995: Deprivation and NHS Region Studies in Medical and Population Subjects No 61 London: The Stationery Office

[bib6] Kim HJ, Fay MP, Feuer EJ, Midthune DN (2000) Permutation test for joinpoint regression with applications to cancer rates. StatMed 19: 335–351, (correction: 2001; 20:655) 10.1002/(sici)1097-0258(20000215)19:3<335::aid-sim336>3.0.co;2-z 10649300

[bib7] Levi F, Lucchini F, Negri E, La Vecchia C (2003) Trends in cancer mortality at age 15–24 years in Europe. Eur J Cancer 39: 2611–2621 14642923 10.1016/j.ejca.2003.03.001

[bib8] National Collaborating Centre for Cancer (2005) Guidance on Cancer Services, Improving Outcomes in Children and Young People with Cancer. London: National Institute for Health and Clinical Excellence

[bib9] Office for National Statistics (2006a) Mortality Statistics: Cause, England & Wales, 2005 Series DH2 No 32 London: Office for National Statistics

[bib10] Office for National Statistics (2006b) Cancer Statistics Registrations. Registrations of Cancer Diagnosed in 2004, England Series MB1 No 35 London: Office for National Statistics

[bib11] Office for National Statistics (2007) Population Estimates for UK, England and Wales, Scotland and Northern Ireland, http://www.statistics.gov.uk/statbase/Product.asp?vlnk=601&More=N accessed 23 February 2007

[bib12] Office for National Statistics (2004) 1981–2001 ONS ICD9–10 Mortality Data Conversion. Version 1.1, CD-ROM

[bib13] Quinn M, Babb P, Brock A, Kirby L, Jones J (2001) Cancer Trends in England and Wales 1950–1999 Office for National Statistics London: The Stationery Office

[bib14] Quinn MJ, Wood H, Cooper N, Rowan S (ed). (2005) Cancer Atlas of the United Kingdom and Ireland 1991–2000. Studies on Medical and Population Subjects No 68 London: Palgrave MacMillan

[bib15] R Development Core Team (2006) R: A Language and Environment for Statistical Computing. Vienna, Austria: R Foundation for Statistical Computing, ISBN 3–900051–07–0 http://www.R-project.org

[bib16] Rowan S, Wood H, Cooper N, Quinn M (2005) Update to Cancer Trends in England and Wales 1950–1999 Office for National Statistics London: The Stationery Office

[bib17] StataCorp (2005) Stata Statistical Software: Release 9. College Station, TX: StataCorp LP

[bib18] Swerdlow A, dos Santos Silva I, Doll R (2001) Cancer Incidence and Mortality in England and Wales. Oxford: Oxford University Press

[bib19] Wood SN (2006) Generalized Additive Models: An Introduction with R. Boca Raton, FL: Chapman & Hall/CRC

[bib20] World Health Organization (1977) International Statistical Classification of Diseases Ninth Revision, Volume 1 Geneva: WHO

[bib21] World Health Organization (1992). International Statistical Classification of Diseases and Related Health Problems, Tenth Revision Volume 1 Geneva: WHO 3376487

